# Disorders of Arousal: A Chronobiological Perspective

**DOI:** 10.3390/clockssleep3010004

**Published:** 2021-01-21

**Authors:** Greta Mainieri, Giuseppe Loddo, Federica Provini

**Affiliations:** 1 Department of Biomedical and NeuroMotor Sciences, University of Bologna, 40139 Bologna, Italy; greta.mainieri2@unibo.it; 2 Azienda AUSL di Bologna, 40124 Bologna, Italy; giuseppe.loddo2@unibo.it; 3 IRCCS Istituto delle Scienze Neurologiche di Bologna, 40139 Bologna, Italy

**Keywords:** parasomnia, circadian rhythm, slow wave sleep, NREM sleep, chronotype, sleep deprivation

## Abstract

Non-rapid eye movement (NREM) sleep parasomnias are characterized by motor and emotional behaviors emerging from incomplete arousals from NREM sleep and they are currently referred to as disorders of arousal (DoA). Three main clinical entities are recognized, namely confusional arousal, sleep terror and sleepwalking. DoA are largely present in pediatric populations, an age in which they are considered as transitory, unhabitual physiological events. The literature background in the last twenty years has extensively shown that DoA can persist in adulthood in predisposed individuals or even appear de novo in some cases. Even though some episodes may arise from stage 2 of sleep, most DoA occur during slow wave sleep (SWS), and particularly during the first two sleep cycles. The reasons for this timing are linked to the intrinsic structure of SWS and with the possible influence on this sleep phase of predisposing, priming and precipitating factors for DoA episodes. The objective of this paper is to review the intrinsic sleep-related features and chronobiological aspects affecting SWS, responsible for the occurrence of the majority of DoA episodes during the first part of the night.

## 1. Background

Non-rapid eye movement (NREM) sleep parasomnias, currently referred to as “disorders of arousal” (DoA), are a group of motor manifestations characterized by the occurrence of incomplete awakenings from NREM sleep [1,2]. They encompass three main clinical entities—confusional arousals, sleep terrors and sleepwalking—whose diagnosis is presently based exclusively on clinical criteria [2]. The lifetime prevalence of DoA is 6.9%, as emerged by a recent meta-analysis, with a higher representation in childhood than in adult series [3]. Adult subjects manifesting episodes of DoA often have a previous history of sleepwalking, while de novo incidence of DoA in adults is thought to be less than 1% [4]. Indeed, a strong genetic component has been noted since the earliest descriptions. Retrospective studies and subsequent longitudinal cohorts highlighted that the clinical manifestations of NREM parasomnias tend to have a robust familial co-occurrence, and children with a parental history of parasomnia have a higher risk of manifesting clinical episodes themselves [5,6,7]. Still, there is no known gene identified in families’ pedigrees. The only acquaintance comes from a family with 22 members and 9 affected individuals, in which a linkage to chromosome 20q12-q13.12 was identified. Among the 28 genes from the exonic sequence involved, the most likely candidate was Adenosine Deaminase gene (ADA), a gene associated with the quantity of slow wave sleep (SWS) [8]. Other studies identified a specific DQB1*0501 HLA haplotype associated with a large cohort of subjects with DoA [9,10].

The exact pathophysiological mechanisms and the neuronal networks underlying DoA are complex and not still fully elucidated. Real-time episodes, rarely captured by neurophysiologic studies (a SPECT in one case and intra-cerebral EEG recording in others), demonstrated a co-existence of sleep and wake in different brain areas [11,12]. In particular, wake-like rhythms in sensorimotor and cingulate cortices are responsible for motor and emotional behaviors during DoA episodes, while deep sleep activity in fronto-parietal associative cortices and hippocampus answers for the typical unawareness and amnesia associated with the episodes [1,12,13]. Therefore, a selective activation of thalamocingulate circuits and the persisting inhibition of the other thalamocortical arousal systems have been postulated in DoA [11]. DoA have usually been described to occur in the first third of the night [14,15,16]. The reasons for this circadian preference are thought to be related with the intrinsic structure of the stage 3 NREM sleep (N3) [13]. In fact, even if some episodes can occur from N2, most of the major episodes arise from SWS [17], whose amount is greater in the first third of the night. Moreover, the overall quantity of N3 undergoes a progressive decrease during lifetime, being more represented in children’s nocturnal sleep. In the earliest studies on sleepwalking, the more frequent incidence of DoA episodes in children rather than adult populations led to hypothesize that a constitutional higher amount of SWS could promote the occurrence of the episodes [18]. However, adult series comparing subjects with DoA and healthy sleepers outlined different results. An increased amount of SWS has been ascertained in some studies [19], but not confirmed in others, showing a substantially equivalent N3 quantity between patients and controls [15,20,21,22,23,24]. Conversely, differences in the amount of N3 are detected when comparing DoA with other sleep disorders, such as obstructive sleep apnea syndrome (OSAS) [18,25], sleep-related hypermotor epilepsy (SHE) [22] or the presence of a high number of periodic limb movements (PLMs) [18,25]. However, in these pathological conditions, the high sleep micro-fragmentation precludes the achievement of a stable deep sleep [17] with a decrease rather than an increase in SWS, in comparison to both DoA and healthy subjects. Therefore, the increased quantity of SWS in DoA subjects appears as an insufficient condition to explain the occurrence of the episodes [18].

In some cases, it has been postulated that the persistence of a greater amount of SWS in adults could be linked to a delayed maturational process of sleep, thus resembling that of childhood [18]. In this regard, it is noteworthy that some developmental disorders, such as autism or attention-deficit/hyperactivity disorder (ADHD), seem to predispose for DoA episodes [14,26,27,28]. However, this is not the case of the majority of DoA subjects, both for pediatric and adult series, who do not display any intellectual disability or cognitive deficit, suggesting that DoA episodes could be the tip of an iceberg of an otherwise physiological response to an arousal trigger in predisposed individuals.

Many potential causes have been explored and shown to potentially affect the structure of SWS, leading to the occurrence of clinical episodes in individuals with a predisposition [18]. Indeed, an intrinsic dysfunction of deep sleep could contribute to the manifestation of the episodes [13], including an inability to maintain N3 and an altered response to sleep deprivation, in comparison to healthy subjects [13]. Together with a defective system in preserving a stable deep sleep, an alteration of the arousal process is hypothesized to play another role in the occurrence of the clinical manifestations, which is the reason these conditions are currently referred to with the acronym of DoA [13]. However, it is likely that the interplay between all these aspects contributes to the development of the clinical behaviors. Whereas the neurophysiological mechanisms of an intrinsic dysfunction of SWS and a defective arousal process are still object of investigation, some models have been proposed to categorize the clinical factors implied in the occurrence of DoA.

**Figure 1:**
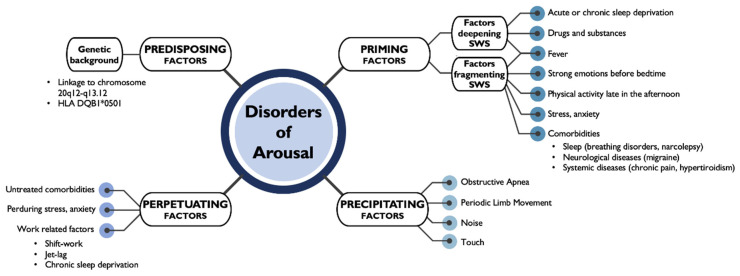
The 4-Ps model of factors involved in DoA pathophysiology. On a predisposed genetic background, different priming factors could lead to the occurrence of DoA episodes, possibly provoked by additional precipitating factors. The persistence of unresolved causes may lead to the perpetuation of DoA in adulthood and even in the elderly. HLA, Human Leukocyte Antigen; SWS, Slow Wave Sleep.

The current model of the 3Ps identifies factors predisposing, priming and precipitating DoA events, the majority of which are directly related with possible rebound or disruption of a probably dysfunctional SWS or to increased arousability due to internal or external triggers [1,18,29]. In cases with no apparent triggers, the episodes arise because of N3 instability, with an increased number of arousals detected by PSG studies [23], an increased cycle alternating pattern (CAP) rate at the micro-structural level [16,30] and a functional alteration of connectivity detected by quantitative analyses of EEG signals [31,32,33]. Moreover, in some adult series, subjects older than 60 years are reported to continue sleepwalking [3], indicating that a fourth P, i.e., perpetuating factors, leading to the persistence of clinical episodes, may be worth investigating (Figure 1).

The purpose of this paper is to review the existing evidence about the occurrence of NREM sleep parasomnias, especially during the first third of the night, and the different factors producing a rebound/increase or a disruption in SWS. These can be either intrinsic sleep-related features or chronobiological factors influencing endogenous sleep rhythms.

## 2. Rebound of Deep Sleep

### 2.1. Acute Sleep Deprivation: Sleep Deprivation Schedules in Laboratory Studies

In healthy subjects, sleep deprivation normally causes an increase in sleep pressure, a rebound and an increased continuity of SWS, together with an increased arousal threshold, resulting in a global decrease in awakenings during sleep [13,18,34]. Conversely, for DoA subjects, sleep deprivation is very often reported from the subject him/herself as a trigger for episodes of particular violence and enough complexity to be recalled and then reported to sleep clinicians (Table 1). In fact, both chronic and acute sleep deprivation have been frequently reported to act as trigger for major episodes in DoA. For this reason, some studies have been specifically designed with the purpose to analyze possible differences or alterations of sleep, in response to sleep deprivation schedules, in subjects with DoA compared to healthy sleepers. In addition, some studies performed only in DoA subjects tried to ascertain if there is an increased propensity for the occurrence of somnambulistic episodes in response to sleep deprivation, with heterogeneous results [20,35,36,37,38].

Some early studies [36] reported that adult sleepwalkers, who had undergone a night of sleep deprivation, had overall improvement in their sleep in their recuperation night, with a decreased sleep fragmentation and no behavioral episodes. The authors hypothesized that an increased sleep pressure might result, in analogy with healthy subjects, in a more stable continuity of SWS. Conversely, Joncas et al. [20], adopting a 38-h schedule of sleep deprivation in 10 DoA patients and 10 healthy subjects, compared their baseline night with their recovery night. Patients with DoA had a significant increase in the number and complexity of behaviors on their recovery night, indicating that sleep deprivation might be a powerful tool to elicit and capture somnambulistic episodes, even in a sleep laboratory setting [20]. Similar results were disclosed by Zadra et colleagues [38], who recorded 30 DoA subjects during a baseline night and after 25 h of sleep deprivation. They disclosed a significant increase in the frequency and complexity of somnambulistic episodes, together with a higher proportion of patients experiencing at least one complex episode, emphasizing that a shorter sleep deprivation protocol may guarantee similar results to longer ones [38]. In another study, the same authors [37] applied a schedule of 25 h of sleep deprivation together with the administration of an acoustic trigger during the recovery night in 10 DoA subjects and 10 controls. The combination of the two factors significantly increased the probability of occurrence of a somnambulistic episode in DoA patients [37]. Taken together, the results from these last studies, even with some differences in samples and design, seem to suggest that different pathophysiologic mechanisms in response to sleep deprivation result in increased fragmentation of SWS in DoA patients and facilitate the occurrence of dissociated episodes [18]. Moreover, the authors suggested that, when recovery sleep is initiated during daytime (a circadian time of increased wake propensity), subjects with DoA may be even more vulnerable to homeostatic sleep pressure, with a significant increase exclusively in SWS awakenings [38].

Clues for explaining this different susceptibility to sleep deprivation in DoA may derive from animal studies [39]. A recent study, targeting the EEG delta power role in sleep homeostatic pressure, both in mice and humans subjected to sleep deprivation, demonstrated two different classes of delta waves, respectively designated as δ1 and δ2. Only δ2 waves were influenced by sleep deprivation, showing a high initial power and a rapid decay within the first hour of the first NREM sleep cycle of recovery sleep. Moreover, in this same time lapse coincident with the deepest NREM sleep, other physiological parameters, such as muscle tone, cardiac activity and temperature, were significantly more similar to wakefulness, suggesting that a phase of “wake inertia” is necessary to recalibrate a normal NREM sleep after sleep deprivation [39]. An exaggeration of this physiological mechanism may lead to the occurrence of episodes in DoA subjects, typically exemplified by a motor and autonomic “wake” activity associated with a cerebral “deep sleep”. In addition, these neurophysiological considerations well suit the empirical evidence of a preferential timing of DoA episodes in the first NREM sleep cycle.

### 2.2. Chronic Sleep Deprivation or Biological Misalignment: Shift or Rotational Working

Some studies performed in the general population analyzed the risk of parasomnias in particular categories, such as nocturnal or rotational workers [40,41]. These individuals may be subject to a chronic sleep deprivation and possible displacement of their own chronobiological rhythm. Other studies specifically targeted these shift-working categories, exploring the prevalence of parasomnias among day, night or rotational workers [42,43].

Among studies conducted in the general population, a study in 15,000 subjects from the US did not disclose a significant increase in nocturnal wandering in shift-working categories [40]. Conversely, in a large epidemiological sample of almost 5000 subjects from the United Kingdom, shift work was significantly associated with confusional arousal, with an odds ratio (OR) of 2.1 [41]. Accordingly, in a study on 2000 nurses questioned about the presence of NREM and REM parasomnias [44] and subdivided into three different groups (day workers, night workers or shift workers -with two or three shifts-), confusional arousals were significantly increased in shift workers compared to both diurnal and night workers. Sleepwalking and sleep terrors were not statistically different in the three work groups. Nevertheless, the increased frequency of confusional arousal in nurses experiencing rotational shifts, in comparison with both “daytime only” and “night time only” workers, suggests that a biological circadian misalignment between the internal circadian clock and the external environment might facilitate the occurrence of DoA episodes [44].

In another study specifically addressing shift-workers (179 offshore workers), a report of sleepwalking was present in 7.5% of night/rotational workers versus 2.3% of day workers [43].

It must be underlined that all these studies relied only upon subjective reports, with no objective tools confirming the diagnosis of DoA, with a consequent possible underestimation of the disorder. The higher prevalence of confusional arousals in comparison to sleep terror or sleepwalking may be related both to a possible underestimation of these disorders and to statistical power implications, due to the lower number of subjects reporting sleep terror or sleepwalking [44]. Finally, it cannot be excluded that subjects with more severe and complex episodes of DoA tend to avoid occupations involving shift-working schedules, with a possible underestimation of their negative effects in predisposed subjects [44].

## 3. Sleep Disruption: Disorders of Arousal

Together with an altered response to sleep deprivation, as the current acronym of DoA suggests, DoA patients have also been described as having a disorder of arousal, characterized by an inability to maintain deep sleep and an increased number of arousals during stage 3 of sleep. Wake and sleep behavior and body homeostasis are controlled by a neuronal network running from the brainstem to the cerebral cortex and working in a unitary fashion according to a caudorostral organization [45]. In particular, the genesis of SWS depends on a complex interplay between the cortex and thalamic nuclei but a considerable effort is still ongoing to identify the specific functions and connections among thalamic nuclei and the different cortical regions [46,47]. In addition, the mechanisms and neuroanatomical substrates of the arousal process during sleep are still matter of debate [48]. The arousal process in sleep has been physiologically considered to have a duplex opposite nature: preserving sleep continuity and ensuring a prompt response to a perturbing stimulus [49,50,51]. In mice, ventromedial thalamic nuclei projecting diffusely to the entire cerebral cortex promotes arousals from NREM sleep [47]. A stereo-EEG study analyzing sleep arousals in humans showed a constant and stereotyped activation of the medial pulvinar nucleus, characterized by the appearance of intermediate EEG frequencies between sleep and wake [50]. One stereo-EEG study showed an abrupt appearance of wake rhythms from delta activity in the ventromedial portion of the thalamus during a confusional arousal [12]. Functional imaging studies in healthy subjects showed that activation and deactivation over different cortical areas, consistent with local sleep/wake activity, is normally associated with the process of arousal per se [49]. This consideration reinforces the idea that episodes of DoA arise from a deregulation of a physiological cortical arousal process, which can have its climax in the clinical motor episode in predisposed individuals, with the consequence that an episode of DoA embodies at the same time the two opposite aspects of the double nature of arousal [33].

### 3.1. SWS Intrinsic Fragmentation

Subjects with DoA have often been assumed to have an intrinsic dysfunction in maintaining a stable SWS [13,18]. This N3 instability encountered in DoA subjects translates into the common polygraphic finding of an increased number of arousals during deep sleep, in comparison to healthy controls. In this regard, an increased number of awakenings during SWS, but not in other sleep phases, has been widely demonstrated in polysomnographic studies [23,52,53]. In particular, two recent works by the same authors validated polygraphic cut-offs to discriminate with good sensitivity and specificity subjects with DoA from healthy subjects [23,53]. They classified the EEG patterns associated with N3 awakenings into three different categories: slow, mixed, and fast arousals. The study in DoA adults [23] revealed that an increased index of SWS awakenings (SWS arousals per hour of N3) and a pattern characterized by slow/mixed arousals are strongly associated with DoA, with a cut-off of 6.8/h and 2.5/h, respectively. These same criteria were also validated in a pediatric population, with different cut-offs [53]. The pattern described as slow arousal is largely corresponding to the A1 phase of CAP, which decreases with age [16,30]. The A1 phase of CAP is considered an anti-arousal pattern, fundamental in younger ages to preserve sleep continuity in a maturational brain process [53]. The CAP rate was previously described to be increased in children [30] and also adults with DoA, especially in the first two sleep cycles [54]. In addition, the A1 phase of CAP has also been assimilated to the hypersynchronous delta waves, frequently described in DoA subjects, especially prior to the clinical episodes [54].

The studies from Lopez et al., adding a quantitative parameter, may improve the accuracy of DoA diagnosis, considering that the latter is presently based only on clinical criteria [23,53]. In future research, it could be interesting to validate Lopez et al. SWS fragmentation criteria not only versus healthy subjects but also versus other sleep pathological conditions, such as sleep breathing disorders or sleep related hypermotor epilepsy, similarly characterized by increased sleep fragmentation.

### 3.2. SWS Microstructural Dysfunction: SWS Distribution, Delta Spectral Power and Sleep Spindles Dynamic

In DoA, microstructural and quantitative EEG signal analyses documented a difference in sleep intensity by the assessment of delta power throughout sleep cycles.

Examining SWS delta power [21] in a study comparing 12 patients with DoA and healthy subjects, a reduction of slow wave activity (SWA) in the first sleep cycle was disclosed. This finding largely corresponded to the visual observation of an increased rate of micro-arousal. Despite the overall decrease in SWA, some seconds prior to the behavioral event an increase in low delta frequencies appeared, visually corresponding to the hypersynchronous delta waves. The authors suggested this increase could be an attempt of the cortex to maintain sleep in response to an arousal activation (the above-mentioned anti-arousal response) [21].

In addition, physiologic sleepers’ deep sleep seems to follow a gradient of SWA power, being highest in the beginning of the night and progressively decreasing throughout the night cycles [19,52], in accordance with the physiological homeostatic process of sleep [55]. In contrast, patients with DoA tend to have a flatter and more homogeneous profile of SWA power throughout the night [19,52].

Moreover, a high-density EEG study highlighted that a decrease in SWA (otherwise meaning an increased activation) over sensorimotor and cingulate areas was consistently and significantly present during NREM sleep (especially SWS), independently of clinical episodes [31]. These findings indicate that, in subjects with DoA, the contemporary presence of local sleep and wake activity is not restricted to the motor episodes but is rather an intrinsic feature of sleepwalkers’ sleep.

**Figure 2:**
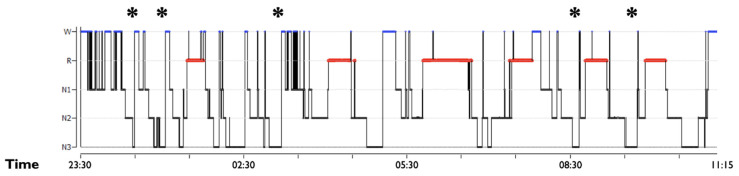
Hypnogram of a patient with DoA. Hypnogram in a 23-year-old DoA patient. The stars (*) indicate the occurrence of DoA episodes during the night. It is noteworthy that N3 is highly fragmented by several awakenings and distributed throughout the night. Some DoA events arise also from the last part of sleep.

A recent study analyzed the *N3 distribution index*, intended as the ((number of N3 epochs in the first half of the night–the number of N3 epochs in the second half of the night)/the total number of N3 epochs of the night) in DoA, SHE patients and controls [17]. The index is significantly increased in control subjects compared with DoA [17], confirming a different distribution of N3 during the night in DoA subjects, with periods allocated in the second part of the night. The consequence of this different distribution is that some clinical episodes do indeed emerge from the second half of the night [19], escaping the general rule of the occurrence of motor episodes only within the first part of the night (Figure 2).

In fact, in addition to the more extensively known and recognizable major episodes, a recent work directed the attention to simple motor episodes, which represent the smaller part of a complex episode, but appear much more frequently during the nocturnal recordings [56]. In a polysomnographic study, the major episodes in DoA were mostly distributed during the first part of the night (confirming literature background), but this was not the case for minor episodes, which were distributed all over the nocturnal NREM sleep [17]. If, on the one hand, this finding confirms the preferential occurrence of major and more complex episodes during the first part of the night, on the other, it opens a scenario in which, even if smaller and clinically unnoticed, the episodes of DoA may occur throughout the night, in line with the homogeneous distribution of SWS throughout the night reported above.

Finally, an analysis of the dynamic of sleep spindles found a reduction of the sleep spindle index in DoA compared to healthy subjects all over the night, but the reduction was statistically significant for the second and the fourth sleep cycle [19]. The reasons for this particular distribution are not clear: a reduced density of sleep spindles may relate to a lower arousal threshold encountered in DoA subjects. Sleep spindles, in fact, through a process of sensory deafferentation, are thought to potentiate sleep continuity, increasing the arousal threshold [57,58].

### 3.3. Fragmentation Due to Sleep and Medical Disorders and Substances

Beyond the presence of an intrinsic sleep dysfunction, sleep disorders contributing to SWS fragmentation may enhance the occurrence of DoA episodes. Apneic events and PLMs, causing fragmentation in sleep continuity, can coexist by chance or trigger DoA episodes [25]. In a large population study, sleep apnea was associated with nocturnal wandering, with an OR of 3.87 [40].

Polysomnographic studies investigating pediatric and adult cohorts of subjects with DoA revealed either a high or a low prevalence of sleep breathing disorders [38,59,60,61,62,63].

Systemic conditions such as febrile illness [64,65], chronic pain, migraine [66] and endocrinological conditions such as hyperthyroidism have been related to the clinical occurrence of DoA episodes [67]. In migraine, a chronic depletion of serotonin leaves place to increased levels of the neurotransmitter during the attacks and a similar mechanism has been theorized in sleepwalking [68,69]. An increased excitability of serotoninergic neurons, producing a release of gross body movements during NREM sleep could induce DoA episodes, as documented in animal models. In rats undergoing microinjections of serotonin in the cholinergic basalis neurons during sleep, wake behaviors associated with an enhancement of slow waves, similarly to somnambulistic episodes, were observed [69].

Stress and anxiety are often considered triggers for DoA occurrence, but systematic studies assessing and relating stress levels with the frequency of episodes are lacking [1].

A wide variety of psychotropic drugs and substances [25] have been reported to induce DoA. In particular, the use of alcoholic substances, with a known disruptive effect on sleep architecture [70], has often been related to parasomnia occurrence, especially in forensic cases [71], although a recent review seems to challenge this view, due to the lack of direct PSG investigation of alcohol effects in sleepwalkers [72].

On the opposite side, the mainstay of the pharmacological treatment of parasomnias is the use of intermediate- and long-acting benzodiazepines, mainly clonazepam, which can act by reducing SWS or suppressing cortical arousal [73].

## 4. Circadian Rhythm and Chronotype

Data on the relationship between parasomnias and circadian rhythmicity, the related sleep-wake disorders and chronotype are scarce, leaving this field largely unexplored. In some studies, in adult subjects investigating chronotype and possibly related sleep disorders, subjects with parasomnia were even excluded [74].

Some evidence comes from pediatric studies by means of questionnaires on sleep disorders and parental reporting. Some of these studies, devoted to children with a developmental disorder, disclosed both a circadian rhythm disorder and DoA episodes [75]. In studies comparing children with ADHD and healthy controls, an evening chronotype was prevalent in patients, with no significant difference from controls in parasomnia subitems [76]. An evening chronotype in children of pre-scholar and scholar age revealed a higher association with several sleep disorders, but no specific association with parasomnia [77,78]. Conversely, an association between a delayed sleep–wake phase and the occurrence of NREM parasomnias was found among Japanese students [79].

In adult populations, an unspecified circadian rhythm sleep time disorder correlated with frequent nocturnal wandering (more than two times per month), with an OR of 3.37 [40] in a US population. No specific correlation was highlighted between circadian sleep rhythm disorders and parasomnia in an epidemiologic survey of 2000 subjects in the Netherlands [80].

Only one study specifically explored the association of chronotype in 150 students with a previous or current history of parasomnia by means of self-administered questionnaires [81]. A total of twenty-one subjects (14%) reported sleep terrors; 11 (7.4%) reported a past history of sleepwalking, with only three subjects reporting active episodes. Neither sleep terrors nor sleepwalking were correlated with a specific chronotype.

In contrast with DoA studies, the relationship between circadian rhythmicity, sleep-wake disorders and chronotype has been widely explored in psychiatric disorders [82,83,84]. Circadian rhythm disruption and the evening chronotype have been associated with bipolar disorder and depression and anxiety scales [84,85]. Subjects with DoA, compared with healthy controls, also report higher scores in depression and anxiety questionnaires [86], together with the presence of a possible history of traumatic events [87]. For these reasons, a systematic investigation of chronotype and circadian rhythmicity in DoA populations could help to shed light on these still poorly known aspects of these fascinating disorders.

Finally, to better evaluate the circadian preferential timing of episode occurrence, it would be interesting to assess the response to melatonin in subjects with DoA, but open trials are lacking [73]. The little available evidence is based on anecdotal reports, in which melatonin was administered in children with a neurodevelopmental disorder. In a single case, with a delayed sleep phase disorder, melatonin-controlled episodes of sleepwalking and sleep terror, but probably as a result of the correction of the circadian disorder and the consequent sleep deprivation. A trial with 5-tryptophan in children induced improvement in DoA episodes through the stabilization of sleep micro-structure and a modulation of the arousal threshold [73].

## 5. Conclusions and Future Directions

In conclusion, NREM parasomnias appear as a condition with a prominent genetic trait, leading to an intrinsic dysfunction in SWS structure associated with susceptibility to possible precipitating factors and with possible lifetime perpetuation. The episodes mostly occur during the first sleep cycles, in accordance with a more dysfunctional N3 during this nighttime. Still little is known about the possible influence of circadian rhythms in this preferential timing of the occurrence of DoA.

Further studies involving, for example, actigraphy assessment of chronotypes and sleep-wake circadian disorders, melatonin level and peripheral clock gene expression are needed, in order to deepen the knowledge and improve DoA clinical management. Actigraphic studies associated with clinical reports, already performed in REM sleep behavior disorder [88,89], could be useful to assess a possible role of circadian rhythm dysregulation both in adults and in children with DoA.

## Figures and Tables

**Table 1:** Clinical and sleep-related features of the different DoA subtypes.

	Confusional Arousal	Sleep Terror	Sleepwalking
General features	Individuals may sit up in bed, looking around with a confused gaze	Individuals sit up abruptly in bed, with intense fearful expression, screaming or defensive gestures	Individuals may walk in the bedroom, with unpurposeful searching behaviors or engaging in more complex actions
Behaviors			
Routine behaviors that occur at inappropriate times	Eye opening, face rubbing, staring, scratching, yawning, searching behaviors	Inconsolable sobbing, anguished face	Calm, quiet ambulation, getting ready for school, driving, moving furniture
Inappropriate or nonsensical behaviors	Manipulating EEG equipment or bed blankets	Recoiling from something, spasmodic searching, evasive behaviors	Walking naked on the roof of the garage or sitting in front of a turned-off TV
Dangerous or potentially dangerous behaviors	/	Hitting, kicking, falling from the bed	Climbing on a chair, jumping out from a window
Sounds	Mumbling, moaning, single words	Screaming, fearful phrases	Sleeptalking
Clinical consequences	Minimal	Possible self injuries	Minimal to severe
Forensic implications	+	++	+++
Time of the night	Usually first third of the night or throughout the night	First third of the night	First third of the night
Sleep Stage			
N3	+++	+++	+++
N2	+	+	+
Sleep features	Increased N3 fragmentation	Increased N3 fragmentation	Increased N3 fragmentation
EEG	Co-existence of anterior delta waves and posterior wake rhythms	Mostly masked by movement artifact	Mostly masked by movement artifact
Autonomic activation	++	+++	++
EMG activation	+	+++	+++
